# The Woman’s Peril; the Man’s Sin

**Published:** 1904-08

**Authors:** 


For Texas Medical Journal.

### The Woman's Peril; The Man's Sin.

LETTER FROM DR. JOSEPHINE KINGSLEY.

In these progressive days, when medical science has worked out so many knotty problems, found a panacea for so many of the ills of life, that the one of most vital importance, and which has caused and is causing more disease and suffering than any, yes, all others together, should so long remain unnoticed and unchecked, is most surprising. I mean the so-called "social evil." The movement of the twentieth century is upon us, and it is a move of self-control.

To whom should parents and children go for advice as to sanitary and physiological conditions necessary to insure health and happiness, if not to the physician.

It is very evident that perfect manhood or womanhood can not be reached through gratification of lust any more than perfect digestion can be secured by the same processes.

The doctrine that self-control is not necessary or possible with the male human being is the one that has brought about present conditions. The medical profession should lead in this oncoming social uplift, or it will lose much that should belong to it.

There is no question of such vital importance to home and country, and no disease so fraught with evil and death as those resulting from these excesses; and all, or much at least, because of no proper teaching in the matter. There should be in every normal human being brain power sufficient to make them capable of self-control. When statistics tell us that 90 per cent of all men are at some time sufferers from gonorrheal affections, we can but see that only 10 per cent of men are physiologically well-developed beings, for normal healthy conditions do not beget disease. Add to this picture the entail that must result and we have a picture that would appall the world if spread out before it, which it will be, for the public are becoming aware that there is a great wrong somewhere.

Are the following conditions necessary evils? There are 300,000 women in our country leading lives of shame in houses of ill-fame, and one-half million candidates for this disease. Though the average life of these women is only five years, it is that much too long for the ruin they cause. No war was ever so devastating in its ravages. Out of 58,000 blind children in our large cities,

15,000 innocent children are blind from gonorrheal ophthalmia alone. Other statistics, no less appalling, can not be given here; but can be found by referring to the *American Journal of Obstetrics and Diseases of Children* for February.

Medical science has studied the removal of the disease without reaching out to the cause, and have failed.

Women are becoming aroused as to the sudden death or invalidism of so many young innocent women so soon after marriage, and are inquiring into the cause.

Dr. Joseph Price says: "There are more and better reasons for locking up in jail a man with gonorrhea than a murderer, and yet they are secreted by the thousands and go on in their murderous work of infection. Can we not remove from our midst or reduce at least many of our most malignant diseases by preventing this culture ground from becoming a predisposing cause?"

How much longer shall we hold our position as a proud American people, with this incubus clinging to 90 per cent of our male population?

An eminent divine has said recently, "A man has no more right to poison his child with a loathsome, an incurable disease than he has to poison it with noxious drugs." Though self-control may never have been a quality listed in his studies or his actions in the past, it is to be hoped the time is not far distant when it may be made a necessary requirement.

San Antonio, Texas, June 25, 1904.

